# Multiple Sclerosis: Pathogenesis and Treatment

**DOI:** 10.2174/157015911796557911

**Published:** 2011-09

**Authors:** Ingrid Loma, Rock Heyman

**Affiliations:** Department of Neurology, University of Pittsburgh Medical Center, Pittsburgh, PA 15213, USA

**Keywords:** Multiple sclerosis, pathogenesis, immune response, interferon-beta, glatiramer acetate.

## Abstract

Multiple sclerosis (MS) is a chronic inflammatory autoimmune demyelinating disease of the central nervous system. It affects approximately 400,000 people in the United States and onset is usually during young adulthood. There are four clinical forms of MS, of which relapsing remitting type is the most common. As the etiology of MS is unknown, finding a cure will remain challenging. The main mechanism of injury appears to be inflammation and 8 agents are now FDA approved to help control MS.  These agents for relapsing forms of MS target different parts of the immune system, with the end goal of decreasing and avoiding further inflammation. No agents are FDA approved for the primary progressive version of MS. FDA approved agents include four preparations of interferon β (Avonex, Rebif, Betaseron and Extavia), glatiramer acetate (Copaxone), mitoxantrone (Novantrone), natalizumab (Tysabri) and fingolimod (Gilenya). There are several drug undergoing phase II and III trials. The heterogeneity of the MS disease process, individual patient response, and medication toxicities continue to challenge the treating physician.

## INTRODUCTION

Multiple sclerosis (MS) is a chronic inflammatory autoimmune demyelinating disease of the central nervous system. Multiple sclerosis affects approximately 400,000 people in the United States alone, most of them being young adults [1]. It expresses itself in four clinical forms: relapsing remitting MS (RRMS), secondary progressive MS (SPMS), primary progressive MS (PPMS), and progressive relapsing MD (PRMS) (See Table **1**). Approximately 87% of patients present with RRMS, characterized by acute attacks (relapses) followed by partial or full recovery (remission) [2]. Patients can manifest with a heterogeneous group of symptoms including changes in vision (unilateral visual loss, diplopia), weakness, dyscoordination, sensory loss or distortions, or changes in bowel and bladder function. Less diagnostic but also disabling symptoms include cognitive change, fatigue, and mood disturbance. Progression of disease may eventually lead to severe disability. Many medications and other measures may be used to ameliorate MS symptoms. The availability of disease modifying therapies has revolutionized the care of patients with the relapsing forms of this disease. These medications help control the underlying disease process, probably by decreasing immune mediated inflammation. They do not cure the disease or reverse the damage that has occurred with prior events. In general the effects of these agents appear more potent when they are given to patients before more severe widespread damage and disability have occurred. As the number of FDA-approved therapies continues to increase and other investigational and off label uses expands, it is helpful to review both the pathogenesis of MS and the effects of the pharmacologic agents. 

## PATHOGENESIS OF MULTIPLE SCLEROSIS

Inflammation of central nervous system is the primary cause of damage in MS. The specific elements that start this inflammation are still unknown. Studies have suggested that genetic, environmental and infectious agents may be among the factors influencing the development of MS. Many immunological studies have been done on the animal model for human MS known as the experimental autoimmune encephalomyelitis (EAE). Based on this model and observations of MS in humans, roles of several immunological pathways involved in MS are being explored. To understand these pathways it is important to first understand some basic points of the immune system in MS. While we have learned much about the immune system by the study of EAE, our lack of understanding of the differences between EAE and MS as well as the complexity of MS (and likely different immunologic subtypes of MS) must be kept in mind when reviewing experimental and immunologic data [3].

There are two general types of immune response. These are the innate and adaptive immune responses. The innate immune response is initiated by microbial products that activate specific receptors, mainly toll-like receptors (TLRs) in an antigen nonspecific manner. Activation of specific subsets of TLR is done by pathogen-associated molecules that are unique for different groups of pathogens. Binding of these molecules to TLRs results in the production of cytokines that modulate the adaptive immune response [4]. The innate system plays a role both in the initiation and progression of MS by influencing the effector function of T and B cells [4]. For example, when activated through TLRs, dendritic cells (DCs) become semi- mature and induce regulatory T cells to produce inhibitory cytokines such as IL-10 or TGF-β [5]. As the dendritic cells continue to mature, they start to polarize CD4^+^ T cells to differentiate into either Th1, Th2 phenotypes or Th17 phenotypes. When T cells differentiate to a Th1 phenotype, inflammation is promoted. Recent studies in the EAE model suggest that glatiramer acetate may induce type II monocytes which promote Th2 cell production and development of regulatory T cells, which then decrease inflammation [6]. 

The adaptive response is initiated by the presentation of a specific antigen to T lymphocytes by antigen presenting cells (APCs). These antigen presenting cells include B cells, dendritic cells, microglia and macrophages. The interaction between the APC and T cell is a chief component to initiate the adaptive immune response. Several types of T cells including CD4^+^ and CD8^+^ phenotype can be activated by APCs. Th1, Th2 cells and Th17 are CD4^+^ effector cells that are polarized in response to exposure to specific interleukins. Once polarized to Th1, Th2 or Th17, these effector T cells secrete specific cytokines. The cytokines produced by Th1 cells are proinflammatory cytokines such as interferon gamma, whereas Th2 cells secrete anti-inflammatory cytokines such as IL-4 and IL-13. Th17 is a newly recognized CD4^+^ T cell subset that produces IL-17, IL-21, IL-22 and IL-26. Like Th1 cells, Th17 cells promote inflammation in MS. IL-17 receptors are seen in acute and chronic MS plaques. Moreover, studies in IL-17 deficient mice show reduction of clinical severity [7]. Th1 cells and Th17 cells migrate to the central nervous system after being primed in the periphery. Subsequent demyelination and axonal loss is then seen [4].

Regulatory T cells (T reg) are another CD4^+^ T cell type involved in the pathogenesis of MS. The role of T reg cells is to regulate effector Th1, Th2 and Th17 cells. The number of T reg cells is the same between MS patients and controls, however patients with MS have reduced T reg function [8]. IFN-β enhances CD4+ regulatory T-cell function [9]. Another study showed that glatiramer acetate can also increase regulatory T cell function by increasing the expression of naïve CD4^+^CD25^+^FoxP3^+^CD31^+^ T cells [10].

Besides the involvement of CD4^+^ T cells in MS pathogenesis, studies have shown that CD8^+^ T cells are present in MS lesions and may have regulatory function in the progression of disease. CD8^+^ cells mediate suppression of CD4^+^ T cell proliferation through the secretion of perforin, which is cytotoxic on CD4^+^ T cells, leading to their inactivation [5]. Also, CD8 ^+^cells kill glial cells, leaving axons exposed [5]. Moreover, CD8^+^ T cells transect axons, promote vascular permeability and activate oligodendrocyte death [5]. All of these events are seen in MS lesions. 

In addition to T cells, B cells and their products are involved in the pathogenesis of MS. It has long been recognized that B cells become plasma cells that make antibodies. The presence of these polyclonal antibodies in the cerebrospinal fluid of MS patients is known as oligoclonal bands. The target of these antibodies is not yet known. Besides the production of antibodies, B cells are now known to produce proinflammatory (lymphotoxin, TNF-alpha produced by memory B cells) and anti-inflammatory (IL-10) cytokines (produced by naïve B cells) [11]. CD20, the target of monoclonal antibody rituximab, is present throughout the maturation of B cells, however it is absent on plasma cells. Moreover, Magliozzi *et al.* [12] showed that B cell follicles are found in the brains of MS patients. These follicles express CD20. Studies have shown the benefits of depletion of CD20^+^ B cells after rituximab infusion, thus inhibiting B cell antigen presentation functions [13]. 

Genetic factors influence MS pathogenesis susceptibility. Studies of families and twin have shown a 40-fold increased susceptibility among first degree relatives of MS patients, suggesting a genetic basis. The HLA locus on chromosome 6p21 which contains the DR antigens has been linked to MS susceptibility. An HLA-DRB1*1501-DQB1*0602 haplotype (DR2) has been repeatedly demonstrated in high-risk populations of Northern European descent. Additional susceptibility loci include chromosomes 10p15, 5p13, and 1p36 [14].

Environmental factors, such as exposure to infectious agents as well as sunlight exposure/vitamin D are felt to account for changing risk of MS when a person migrates from one risk area to another before age 15 years old [15]. Many recent studies suggesting an infectious etiology have been conducted in children and adolescents with MS. Among the pathogens possibly involved are human herpes virus type 6, Epstein Barr virus, and mycoplasma pneumoniae [16]. It is speculated that one way for pathogens to produce MS would be by molecular mimicry. The pathogens may have peptides with direct sequence homologies with myelin components. Also, it is clear that common viral infections such as upper respiratory tract infections and bacterial urinary tract infections may trigger MS relapses. The mechanism by which this happens is still unknown. Multiple studies of sunlight exposure and vitamin D levels suggest a potential protective effect of early life sun exposure or vitamin D consumption [17].

## TREATMENT OF MULTIPLE SCLEROSIS

On its website the National MS society listed more than 136 ongoing clinical trials testing different treatments for multiple sclerosis [18]. There are currently 8 FDA approved agents for relapsing forms of MS. No agents are FDA approved for the primary progressive version of MS (See Table **2**). FDA approved agents include four preparations of interferon-beta (*Avonex, Rebif, Betaseron and Extavia*), glatiramer acetate (*Copaxone*), mitoxantrone (*Novantrone*), and natalizumab (*Tysabri*) and the recently approved first oral medication fingolimod (*Gilenya*) [18] (See Table **3**). Many other immunologically active agents are used off label and others are nearing study completion and FDA application. The differing types and durations of immunologic effects of these agents will increase the complexity and likely risks of future MS care. 

### Interferon-Beta

Interferon beta (IFN-beta) was first approved by the FDA for MS treatment on 1993. It has been shown to reduce relapse rate, decrease disability progression, and MRI evidence of disease activity [19]. The clinical efficacy of IFN-beta is greater in RRMS than in SPMS. The exact of mechanism of how IFN-beta affects MS is uncertain, however several potential pathways have been postulated. Among these mechanisms, inhibition of T cell activation and proliferation as well as reduction in matrix metalloproteinase activity may be important. IFN-beta has numerous other immunologic effects. It reduces the production of proinflammatory cytokines and induces the production of anti-inflammatory cytokines by increasing suppressor T cell activity [20]. 

There are four IFN-beta products available on the market. Interferon beta-1b products (*Betaseron *and the identical *Extavia*) are recombinantly produced by Escherichia coli bacteria. It differs from the IFN-beta made endogenously in humans as it has a single amino acid substitution and is not glycosylated. Interferon beta-1b is administered *via *subcutaneous injection every other day and is titrated to a target dose over 6 weeks. Interferon beta-1a (*Avonex* and *Rebif*) are recombinant peptides produced in Chinese hamster ovary cells and are identical to natural human interferon-beta. The *Avonex* formulation is given intramuscularly once a week and the *Rebif* formulation is given subcutaneously 3 times per week. All four formulations bind to the same type I interferon receptor expressed on human cells. Neutralizing antibodies can negate the benefits of these agents [21]. The rate of neutralizing antibody formation varies between the different interferon beta products, with interferon beta 1-b having the highest rate and the once weekly intramuscular interferon beta 1-a having the lowest neutralizing antibody induction rate. A review of the biologic effects of neutralizing antibodies and a suggested clinical approach is discussed in Polman *et al.* 2010 [22].

The most common adverse effects of interferon beta are injection site inflammation, headache, and flu like symptoms (fever, myalgia and rigors), fatigue and possibly depression. Other adverse reactions include lymphopenia, thrombocytopenia, and elevated liver transaminase levels [19,23]. Prior to starting IFN-beta therapy and periodically thereafter patients should have a complete blood cell count and hepatic function tests [23]. 

### Glatiramer Acetate

Glatiramer acetate (GA), formerly known as copolymer 1, is a random polymer of glutamic acid, lysine, alanine and tyrosine, the most common amino acids in myelin basic protein. The exact mechanism of action is not clear yet Racke *et al.* [24] showed that GA inhibits response of various antigen-specific murine T cell hybridomas. It also blunts human myelin basic protein-specific T cell lines from lysing targets in the presence of three human leukocyte antigen-DR types associated with MS. Further studies have shown that GA increases cytokine levels such as IL-10, TNF-alpha, and IL-4, thus altering the cytokine population to a more regulatory type [24]. It also increases the expression of Foxp3 in CD4^+^ CD25^+^T regulatory cells, thus increasing anti-inflammatory action. Therefore it appears that GA shifts the GA-reactive lymphocyte population from a proinflammatory Th1 state to an anti-inflammatory Th2 state. Further, GA has been shown to act upon CD8^+^D T cells by correcting their regulatory deficit [25].

There is one formulation of GA marketed as *Copaxone*, and it is currently approved for the treatment of RRMS. It has proven effects on exacerbation rate and MRI clinical assessment. It is given subcutaneously on a daily basis. Side effects may include erythema, induration, or lipoatrophy at the sites of injections. An immediate post-injection systemic reaction can occur seconds to minutes after injection. The symptoms consist of chest tightness, dyspnea, tachycardia, flushing and palpitations. This reaction lasts 10-20 minutes, and has not been determined to be dangerous to patients [24]. There are no specific blood tests that need to be monitored while the patient is taking GA. 

#### Monoclonal Antibodies 

Monoclonal antibodies (mAbs) have been studied since the 1980's. There are three different types of monoclonal antibodies differentiated by their structural similarity to human antibody structure. Humanized antibodies consist of more than 90% human components with the balance from the original murine structure. These antibodies include natalizumab, alemtuzumab and daclizumab. Chimeric antibodies are at least 66% human structure and structure, and rituximab belongs to this class of antibodies. Fully human antibodies have no murine structural components [26]. Each mAb is developed to bind to a specific target molecule. mAb mechanism of action depends on the distribution of the targeted molecule, efficacy of the antibody in reaching the target, the interaction between the antibody and target, and the effector functions of the interaction. The mAbs may interact with their target by binding, blocking or signaling [26]. The type of interaction depends on its Fab activity. A binding mAb can mark a target for destruction through is effector function or through conjugation of the mAb to a toxin. Another mechanism is by blocking the epitope needed for ligand interaction, thus preventing signaling. Both natalizumab and daclizumab are blocking mAbs. Monoclonal antibodies can also mediate cytotoxic immune responses and destroy targeted cells. This effector function depends on the Fc domain of the antibody. To date the four monoclonal antibodies most studied for MS are natalizumab, daclizumab, alemtuzumab and rituximab [26]. 

### Natalizumab

Natalizumab (*Tysabri*) is a humanized monoclonal antibody with an IgG4 framework. It was specifically designed for the treatment of MS and was FDA approved on 2004. It was temporarily withdrawn from the market in 2005 after several cases of fatal progressive multifocal leukoencephalopathy were reported in patients treated with natalizumab. It was reapproved on 2006 as a monotherapy for the treatment of RRMS [27]. Its target molecule is CD49, the α4 subunit of very late antigen-4 (VLA-4) receptor. VLA-4 interacts with vascular cell adhesion molecule-1 so that immune cells can migrate through the blood brain barrier. By binding to CD49, natalizumab prevents the adhesion between the endothelial cell and the immune cell, thus migration of leukocytes into the central nervous system is blocked [27]. This agent has robust benefits on relapse rate, disability progression, and MRI activity. The risk of PML requires clinical surveillance for infection with JC virus while treated with this agent.

### Alemtuzumab

Alemtuzumab (*Campath-1H*) is a humanized antibody that was initially approved for the treatment of B-cell chronic lymphocytic leukemia. It remains an investigational agent for MS. Its target molecule is CD52, a glycoprotein expressed widely throughout on T and B cells, natural killer cells, dendritic cells, monocytes, macrophages and granulocytes with the exemption of neutrophils [28]. Alemtuzumab causes a complete depletion of CD52 bearing cells. It depletes cells that mediate Ab-dependent cellular cytotoxicity, i.e. natural killer cells. Studies have shown that the depletion of these immune cells is associated with a decrease in contrast enhancing lesions in MS, thus suggesting stabilization of the blood brain barrier [28]. A phase II clinical trial comparing the efficacy of alemtuzumab versus interferon beta-1a in patients with RRMS showed a 71% reduction in the rate of sustained accumulation of disability and 74% reduction of relapse rate in patients treated with alemtuzumab compared to patients treated with interferon beta-1a [29]. Despite its efficacy, a number of potentially dangerous adverse effects and complications may occur in patients treated with alemtuzumab and limit its use. Such side effects include idiopathic thrombocytopenic purpura, Graves' disease and Goodpasture syndrome, all which are Ab-mediated autoimmune diseases [29]. 

### Rituximab

Rituximab (*Rituxan*) is a chimeric murine/human IgG1 monoclonal antibody. Its target is CD20, an antigen produced only on mature B cells and not on the Ab-producing plasma cells. This monoclonal antibody is FDA approved for the treatment of rheumatoid arthritis and B cell lymphoma and it remains an investigational agent for treatment of MS. Rituximab's primary mechanism of action is a complete depletion of B cells [30]. This depletion of B cells occurs within 2 weeks of initiating treatment and can last for more than 6 months. The exact mechanism of benefit with rituximab treatment remains uncertain. Cases of PML have occurred after the use of this agent as well [31]. 

### Daclizumab

Daclizumab (*Zenapax*) is a humanized monoclonal antibody with an IgG1 framework. It was used initially for the prevention of allogenic tissue transplantation. Its target molecule is CD25, the IL-2 binding epitope of the α-chain of the IL-2 receptor [32]. IL-2 plays an important role both in the regulation of lymphocyte expansion and contraction. 

## CYTOTOXIC AND OTHER AGENTS

Clinicians may use a variety of cytotoxic agents for control of MS despite only mitoxantrone having FDA approval for MS. Off label use of other cytotoxic agents is based on smaller studies, and agents most frequently used include cyclophosphamide, azathioprine, methotrexate, and mycophenolate mofetil [33]. The main mechanism of action of all these agents appears to be a more broad immunosuppressive action and these agents not unexpectedly may increase the risk of infections and neoplasia. Other immunomodulatory agents used include intravenous immunoglobulin and corticosteroids. Overall the risks of cytotoxic drugs for treatment of multiple sclerosis are proportional to the length and intensity of immunosuppression. Data regarding their efficacy as monotherapy or in combination with FDA approved immunomodulatory is still limited. Thus, cytotoxic drugs are not the first line agents used in the treatment of MS. 

### Mitoxantrone

Mitoxantrone (*Novantrone*) is a synthetic anthracenedione that intercalates into DNA. It causes cross-linking and strand breaks and inhibits topoisomerase II, thus interfering with DNA repair. Besides causing generalized immunosuppression, mitoxantrone inhibits monocyte and lymphocyte migration, induces apoptosis of dendritic cells, decreases the secretion of proinflammatory cytokines such as tumor necrosis factor, interleukin-2 and interferon-g [33]. It also inhibits B cell function, increases T cell suppressor function and inhibits macrophage mediated myelin degradation. Mitoxantrone was approved by the FDA on 2000 for SPMS and worsening RRMS [33]. It can be given intravenously every 3 months. Side effects include bone marrow suppression, mild alopecia, nausea and possible transient bluish discoloration of the sclera and urine [34]. Risks also include vacuolar cardiomyopathy, treatment related leukemia, and sterility/teratogenesis [34]. 

### Cyclophosphamide

Cyclophosphamide is a synthetic chemical agent related to nitrogen mustards. It is transformed in the liver to active alkylating metabolites to then cross link DNA. In addition to generalized suppression, cyclophosphamide increases Th2 cells and targets selectively CD45^+^/CD4^+^/RA T cells [33]. Its side effects include alopecia, nausea, vomiting, amenorrhea, myelosuppression, hemorrhagic cystitis, bladder carcinoma and other secondary cancers, and sterility/teratogenesis [35]. Currently, cyclosphosphamide is used off label for RRMS. 

### Azathioprine

Azathioprine is a purine antagonist that blocks the de novo pathway of purine synthesis, thus depleting the production of lymphocytes that lack a salvage pathway. Azathioprine is sensitive to inactivation by thiopurine S-methyltransferase polymorphisms [33]. Besides immunosuppression, the side effects include gastrointestinal and hepatic toxicity. 

### Methotrexate 

Methotrexate inhibits the synthesis of DNA, RNA and protein by inhibiting dihydrofolic acid reductase, which is necessary for the synthesis of thymidylate. Through this reaction, lymphocyte production is inhibited, thus reducing inflammation. Side effects include myelosuppression, gastrointestinal toxicity, pneumonitis, and hepatic fibrosis. This agent is most often used orally in lower doses but has also been tried in high dose intravenous regimens. The Cochrane study done by Gray *et al.* [36] showed that methotrexate had a non-significant trend in reduction of sustained EDSS progression or number of relapses in progressive MS. Currently methotrexate is used only off-label for RMMS. 

### Mycophenolate Mofetil

Mycophenolate mofetil causes lymphocyte depletion, inhibits T cell activation and B cell function, and inhibits B and T cell migration through the blood brain barrier. Its mechanism involves the inhibition of inosine monophosphate dehydrogenase activity, which is needed for purine synthesis [33]. Side effects include leukopenia, anemia, headaches and diarrhea.

### Intravenous Immunoglobulin

Treatment with intravenous immunoglobulin (IVIG) has been reported as beneficial for treatment of patients with RRMS. A meta-analysis study done by Sorensen *et al.* [37] showed that patients treated with monthly doses of IVIG had significant decrease on relapse rate, improved disability scores and decrease deterioration. The dose ranged widely from 0.2 mg/kg to 2 g/kg. No ideal dosage was determined. Another study done by Fazekas *et al.* [38] showed that patients treated with IVIG (0.2-0.4 mg/kg) did not show significant improvement or relapse rate or disability compared to patients with placebo. The number of patients in each arm of this study was relatively small however and as the data on IVIG remains ambiguous it is not usually used as a first line treatment. It might be considered in patients who are pregnant, [39] cannot tolerate FDA approved therapies, or in patients with frequent infections. 

### Corticosteroids

Corticosteroids inhibit lymphocyte proliferation and the synthesis of most pro-inflammatory cytokines. Because of their potent anti-inflammatory effects, corticosteroids have been used to treat MS for the last 50 years. Today corticosteroids are standard treatment for patients with acute relapses, however it is not yet clear if they are as efficacious as a long term treatment. Ciconne *et al.* [40] did a Cochrane review to study the long term effect of corticosteroids in patients with all types of MS. The analysis showed that overall there was no clear evidence that corticosteroids slowed or reversed disease progression. One study used periodic high dose methylprednisolone and showed a significant reduction in the risk of disability progression and brain atrophy at 5 years in patients with RRMS [41]. Because of the heterogeneity of the study results, it was concluded that there was not enough evidence to prove corticosteroids prevent disease progression. The acute and long term risks of corticosteroids are well known and may be serious. 

### Emerging Treatments

To date all the FDA approved treatments for MS are given *via *injection or infusion. A significant amount of research has been done to develop oral agents in the hope of patient compliance and tolerability. Fingolimod is a new oral medication with a recently approved FDA application and is awaiting wider use [42]. A recent placebo controlled trial was done using cladribine in patients with RRMS [43]. Ongoing clinical trials are being conducted with three other oral agents: laquinimod, fumarate (BG00012) and teriflunomide [44]. 

### Fingolimod

Fingolimod (FTY720) is a structural analogue of sphingosine. It targets receptors of sphingosine-1-phosphate (S1P). S1P is an important signaling lipid involved in the migration of lymphocytes from secondary lymphoid organs to the periphery. Studies have shown that with higher density of S1P, there are more lymphocytes released from secondary lymphoid organs [44]. Other studies have shown that FTY720 inhibits mature dendritic cells migration to secondary lymphoid organs [45]. FTY720 also improves myelination in animal models of EAE. Recently, a double blind study done by Cohen *et al.* [42] compared the efficacy of oral fingolimod (0.5-1.25 mg) to intramuscular interferon beta-1a in patients with RRMS. A total of 1153 patients were studied. The results showed that patients treated with fingolimod had a lower annualized relapse rate (0.20) compared to patients treated with interferon (0.33). Moreover, MRI findings supported the clinical results. There was no difference with respect to progression of disability between the two groups. Risks include transient cardiac conduction changes as well as potentially severe viral infections [42]. 

### Cladribine

Cladribine (2-chlorodeoxyadenosine) is an adenosine deaminase-resistant purine nucleoside. Cladribine induces apoptosis of lymphocytes by increasing deoxynucleotides levels in these cells [46]. This immune cell depletion results in decrease of inflammation. Cladribine causes sustained reduction in CD4^+^ cells and CD8^+^ T cells and transient reduction in CD19+ B cells, without affecting other immune cells [43]. In a recent study by Giovannoni *et al.* [43] patients with RRMS were randomly assigned to three groups who received either 3.5 mg/kg or 5.25 mg/kg of cladribine or placebo. The patients who received either 3.5 mg/kg or 5.25 mg/kg of cladribine had a significant lower annualized relapse rate (0.14 and 0.15 respectively) compared to the placebo group (0.33). Moreover the cladribine group had a significant reduction in MRI evidence of MS activity. Adverse effects included lymphopenia (21.6% in the 3.5 mg/kg group and 31.5% in the 5.25 mg/kg group vs. 1.8% in the placebo group), and herpes zoster (8 patients in the 3.5 mg/kg group and 12 patients in the 5.25 mg/kg groups vs. none in the placebo group. An initial FDA review did not result in an approval and additional data submission is underway.

### Laquinimod

Laquinimod is a derivative of immunomodulatory agent linomide (quinoline-3-carboxamide). Its target has not been identified yet. Studies have shown that laquinimod induces the release of TGF-beta and favors Th2 cytokine production. Preliminary studies have shown that laquinimod decreases MS disease activity without causing generalized immunosuppression [45]. Study completion and FDA application is expected in 2011.

### Fumarate

Fumarate (BG0012) is an immunomodulatory agent whose exact mechanism of action is unknown. Studies have shown that it activates the nuclear factor-E2-related factor 2 (Nrf2) transcriptional pathway [47]. A current phase II clinical trial has shown that fumarate decreases the number of new gadolinium-enhancing lesions on MRI by 69% compared to placebo (p<0.0001) [48]. 

### Teriflunomide 

Teriflunomide is an active metabolite of leflunomide. It is currently approved for the treatment for rheumatoid arthritis. Its mechanism of action consists on inhibiting dihydroorotate dehydrogenase, which is a rate limiting step in pyrimidine synthesis [49]. Other actions include suppression of pro-inflammatory cytokines, inhibition of tyrosine kinase activity and inhibition of interaction between APCs and T cells [49]. A phase II clinical trial showed a decrease on active lesions in treated RRMS patients compared to placebo by 56% (p<0.001) [50]. 

## CONCLUSION

The unknown etiology, probable disease heterogeneity, and immune system complexity will continue to provide challenges for clinicians treating MS. To date there is no cure for MS, and medications which decrease immunologic functions may have significant risks. The short term efficacy and safety of newer agents is being explored however the long term risks of these agents, particularly when used in combination or succession will remain uncertain. Moreover, physicians are faced with the treating potentially pregnant woman and at times even children. MS is more common in young adults, and females are predominantly affected. Also, recent studies show that MS can occur in children. Up to 5% of patients diagnosed with MS in the United States per year are children [51]. The use of a national MS disease registry may assist with the better understanding of both efficacy and safety as the treatment options expand. Only after the etiology of MS is better understood will more focused, effective, safe and permanent treatments arise.

## Figures and Tables

**Table 1:** Types of Multiple Sclerosis

Type	Disease Course
Relapsing/Remitting Multiple Sclerosis (RRMS)	Most common type, accounts for approximate 85% of cases. Characterized by discrete attacks that evolve over days to weeks followed by some decree of recovery over weeks to months. In between attacks, the patient has no worsening neurological function.
Secondary Progressive Multiple Sclerosis (SPMS)	Characterized by initial relapses, followed by gradual neurological deterioration not associated with acute attacks.
Primary Progressive Multiple Sclerosis (PPMS)	Characterized by steady functional decline from the onset of the disease. No relapses ever.
Progressive Relapsing Multiple Sclerosis (PRMS)	Characterized by steady functional decline from onset of the disease with later superimposed acute attacks. PRMS and PPMS cannot be distinguished during early stages, until the relapses occur

**Table 2:** Overview Treatment for Different Types of Multiple Sclerosis

Type of Multiple Sclerosis	Treatments
Non-symptomatic Multiple Sclerosis	Interferon beta -1a and 1b, glatiramer acetate, intravenous immunoglobulin
Relapsing Remitting Multiple Sclerosis and Secondary Progressive Multiple Sclerosis	Interferon beta-1a and 1b, glatiramer acetate, natalizumab, intravenous immunoglobulin, pulse methylprednisolone (for acute relapses). If poor response: mitoxantrone, cyclosphosphamide, mycophenolate, azathioprine, methotrexate, methylprednisolone, intravenous immunoglobulin, cyclosporine
Primary Progressive Multiple Sclerosis and Progressive Relapsing Multiple Sclerosis	No specific treatment has been shown to be effective. Ineffective agents: interferon beta, mitoxantrone, rituximab

**Table 3:** Multiple Sclerosis Drug Treatment Dosages

Drug	Dosage Examples
Interferon beta	
Interferon beta-1a (Rebif)	22 mcg or 44 mcg subcutaneous three times a week (initial titration)
Interferon beta-1a (Avonex)	30 mcg intramuscular once a week (initial titration)
Interferon beta-1b (Betaseron & Extavia)	250 mcg subcutaneous every other day (initial titration)
Glatiramer acetate (Copaxone)	20 mg subcutaneous once a day
Fingolimod (FTY720;Gilenya)	0.5 mg orally once a day
Monoclonal Antibodies	
Natalizumab (Tysabri)	300 mg intravenous every four weeks
*Rituximab (Rituxan)	1000 mg dosed four times a year
Cytotoxic and Other Agents	
Mitoxantrone	5 to 12 mg/m^2^ intravenous every three months
*Cyclophosphamide	Wide dose range
*Azathioprine	2 mg/kg oral every day
*Methotrexate	7.5 mg to 20 mg once per week
*Mycophenolate mofetil	500 to 1000 mg oral twice a day
*Methylprednisolone (as disease modifying therapy)	500 to 1000 mg intravenous daily pulses, monthly to every 4 months
*Intravenous Immunoglobulin	0.2 to 1 g/kg intravenous every month
FDA Application Pending Medications	
*Cladribine	3.5 to 5.25 mg/kg intermittent pulses

“*”” : off label or pending FDA approval.
